# Application of Intravoxel Incoherent Motion in the Evaluation of Hepatocellular Carcinoma after Transarterial Chemoembolization

**DOI:** 10.3390/curroncol29120774

**Published:** 2022-12-14

**Authors:** Xiaofei Yue, Yuting Lu, Qiqi Jiang, Xiangjun Dong, Xuefeng Kan, Jiawei Wu, Xiangchuang Kong, Ping Han, Jie Yu, Qian Li

**Affiliations:** 1 Department of Radiology, Union Hospital, Tongji Medical College, Huazhong University of Science and Technology, Wuhan 430022, China; 2 Hubei Province Key Laboratory of Molecular Imaging, Wuhan 430022, China

**Keywords:** hepatocellular carcinoma, chemoembolization, magnetic resonance imaging, intravoxel incoherent motion

## Abstract

(1) Background: To assess the efficacy of the quantitative parameters of intravoxel incoherent motion (IVIM) diffusion-weighted imaging for hepatocellular carcinoma (HCC) diagnosis after transarterial chemoembolization (TACE). (2) Methods: Fifty HCC patients after TACE were included and underwent MRI. All of the patients were scanned with the IVIM-DWI sequence and underwent TACE retreatment within 1 week. Referring to digital subtraction angiography (DSA) and MR enhanced images, two readers measured the *f*, D, and D* values of the tumor active area (TAA), tumor necrotic area (TNA), and adjacent normal hepatic parenchyma (ANHP). Then, the distinctions of the TAA, TNA, and ANHP were compared and we analyzed the differential diagnosis of the parameters in three tissues. (3) Results: For values of *f* and D, there were significant differences between any of the TAA, TNA, and ANHP (*p* < 0.05). The values of *f* and D were the best indicators for identifying the TAA and TNA, with AUC values of 0.959 and 0.955, respectively. The values of *f* and D performed well for distinguishing TAA from ANHP, with AUC values of 0.835 and 0.753, respectively. (4) Conclusions: Quantitative IVIM-DWI was effective for evaluating tumor viability in HCC patients treated with TACE and may be helpful for non-invasive monitoring of the tumor viability.

## 1. Introduction

Because of the high prevalence and mortality rate of hepatocellular carcinoma [1], many treatment options are continuously being explored and attempted in order to prolong the survival of patients. In the last several years, despite the advancement of diagnostic and therapeutic techniques, the high rate of postoperative metastasis and recurrence still results in a poor prognosis [2,3,4]. In China, the burden of disease caused by HCC is increasing, and there was no significant improvement in the overall 5-year net survival rate of patients [5]. Transarterial chemoembolization (TACE) has developed into one of the main treatments for non-removable liver cancer due to its ability to delay the progression of the disease and improve the patient prognosis [6]. 

Repeated TACE is necessary for larger tumors or tumors that fail to respond to the first treatment [7]. Breunig IM’s research showed that patients who received TACE four times lived one year longer than patients who received TACE once [8]. Repeated TACE requires that patients undergo multiple CT or MR examinations to assess the postoperative efficacy, but CT examinations have a larger radiation and the enhancement of the lipiodol deposition area after TACE may be masked and cannot be accurately assessed. MR enhancement can effectively remove the interference of the lipiodol deposition, but there are still some difficulties in the evaluation of tumor viability after TACE treatment. How to accurately assess the tumor response after treatment is very important for the repeated treatment and prognosis of the tumor. In addition to CT or MR enhancement commonly used in clinical practice, quantitative observation indicators may be helpful for the response after treatment.

Fitted intravoxel incoherent motion (IVIM) of multi-b value diffusion-weighted imaging can assess and quantify the diffusion of water molecules in capillary networks and microcirculatory perfusion in the absence of contrast agents by using a bi-exponential model. Multi-b value diffusion weighted imaging of IVIM could evaluate and quantitatively analyze the diffusion of water molecules and micro-circulation perfusion in the capillary network by using a biexponential model in the absence of contrast agents [9]. Studies have shown that the parameters of IVIM are capable of diagnosing prostate cancer [10] and breast cancer [11,12]. IVIM is also widely studied in HCC, such as for the distinction of malignant and benign tumors [13,14] and for the grading of liver cancer [15].

This study aims to quantitatively identify the tumor active area (TAA), tumor necrotic area (TNA), and adjacent normal hepatic parenchyma (ANHP) after TACE with IVIM sequencing, and consequently provide a quantitative and noninvasive monitoring method in clinical practice.

## 2. Materials and Methods

This study protocol was permitted by the Ethics Committee of Tongji Medical College of Huazhong University of Science and Technology and was implemented with the guiding principles of the Declaration of Helsinki. Patients participating in the study signed informed consent.

### 2.1. Patient Demographics

**Figure 1:**
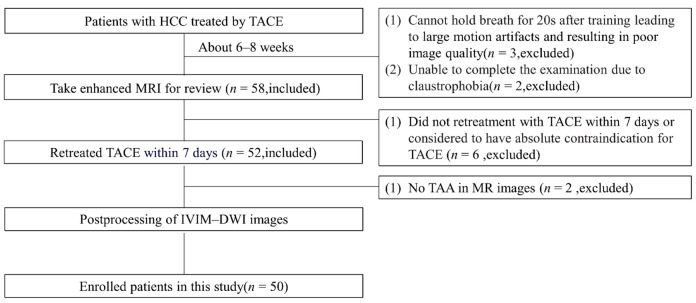
The flowchart shows the inclusion and exclusion process of the study patients. HCC: hepatocellular carcinoma; TACE: transarterial chemoembolization; TAA: tumor active area.

Fifty consecutive patients (9 women and 41 men) with HCC treated with TACE in the past 1–3 months were subsumed. The criteria for patient selection were as follows: (i) diagnosed with HCC [16], (ii) received TACE treatment in the past 1–3 months, and (iii) scheduled to undergo TACE retreatment within 1 week of the review. Of the 63 patients who were assumed to be eligible for this study, 13 patients were eliminated for the following reasons: (i) unable to hold their breath for 20 s after training, leading to large motion artifacts and poor image quality (*n* = 3); (ii) unable to complete the examination due to claustrophobia (*n* = 2); (iii) did not take TACE retreatment within 7 days after MR examination or had contraindication for TACE (*n* = 6); and (iv) no TAA was visible on the MR images or DSA images (*n* = 2). The flow diagram in Figure 1 indicates the process of enrollment for the study population.

### 2.2. MR Examinations

Before the MRI examination, patients were informed of the purpose of the experiment, required to fast for at least four hours, and were trained to hold their breath for 20 s while keeping the degree of inhalation as consistent as possible.

MRI was performed with 1.5 T MRI (MAGNETOM Aera, Siemens Healthineers, Germany) and an 18-channel phased array. The patients were placed head first in the supine position. First, routine MRI sequences were collected, and then functional sequences needed for the study were added (Table 1). The IVIM-DWI sequence was collected under free breathing, and a total of 12 b-values were selected (0, 10, 20, 30, 50, 80, 150, 200, 400, 600, 800, and 1000 s/mm^2^). When the b-value was 0 s/mm^2^, the number of excitations was 1, and when the b-value was 10–1000 s/mm^2^, the number of excitations was 2.

### 2.3. TACE Treatment

Digital subtraction angiography (DSA) was included in the TACE treatment. We compiled patients’ data who needed TCAE in clinical treatment, but did not have additional examinations. The right femoral artery was used for venipuncture using the Seldinger technique under local anesthesia. A 5 F Yashiro catheter and coaxial microcatheter were placed into the celiac artery, the superior mesenteric artery, and the hepatic artery. Then, the contrast agent was injected into the catheter to obtain arterial DSA photography. According to the photography results, the superselective catheter was set in the branch of the intrahepatic tumor artery, and the required drugs were slowly injected for embolization. After the operation, the endotracheal tube was removed, and the wound was bandaged using pressure.

### 2.4. Reference Standard

The LI-RADS treatment response algorithm (TRA) is widely used to evaluate the response of HCC to locoregional treatment. However, LI-RADS is not equivalent to pathological findings, and to make the results more accurate, we combined the LI-RADS and DSA results. Using LI-RADS-TRA based on MR images and DSA images as the reference standards, two radiologists who had more than 8 years of experience in abdominal diagnosis individually evaluated the MR images in accordance with LI-RADS standards [17] and were unaware of the DSA results. TAA was defined as LI-RADS-treatment response (LR-TR) viable. Two interventionalists with over ten years of experience evaluated the DSA images, but were unaware of the MR results. When observing the tumor staining, TAAs were considered to exist. Any disagreements were resolved by consulting a senior radiologist. When the area was evaluated as LI-RADS-treatment response (LR-TR) viable in LI-RADS TRA and observed staining in DSA, it was considered as TAA; TNA areas was selected when it was evaluated as nonviable by both DSA and LI-RADS TRA.

### 2.5. Postprocessing IVIM Images

The Medical Imaging Interaction Toolkit (MITK) Diffusion software (2018.09.99, German Cancer Research Center, Heidelberg, Germany) [18] was used to process the images. The ROIs of TAA, TNA, and ANHP should avoid large vessels whenever possible. The ROIs of TAAs were drawn along the edge of the actual part on the three consecutive layers showing the largest outline of the lesion. TNAs were identified in TACE-treated areas that were consistently unenhanced after multiple calculations in three-phase enhanced images.

The ANHP areas were selected in the normal liver parenchyma area 1 cm away from the tumor for fear of the potential impact of microvascular invasion on the measurement results [16,19]. The corresponding IVIM-DWI parameter values were obtained as follows: perfusion fraction (f), pure diffusion coefficient (D), and pseudo-diffusion coefficient (D*). All of the ROIs were segmented manually and repeated 2 weeks later by an abdominal radiologist with 5 years of experience, and a senior radiologist with 20 years of experience confirmed each ROI.

### 2.6. Statistical Analyses

The measurement results are described as the mean ± standard deviation. Statistical software (GraphPad Prism, version 8, San Diego, CA, USA; SPSS, version 21.0, IBM Corporation, Armonk, NY, USA, and MedCalc, version 15.2.2, MedCalc, Mariakerke, Belgium) was used to execute all of the statistical analyses. The Kolmogorov–Smirnov test was performed on each group of normally distributed data. The intraclass correlation coefficient (ICC) test was used to assess consistency between the two readers. The differences between the TAA, TNA, and ANHP parameters were analyzed using the Kruskal–Wallis H test. *p* < 0.05 was regarded as being statistically significant. The differential diagnostic efficacy of the TAA, TNA, and ANHP parameters was analyzed by drawing a receiver operating characteristic (ROC) curve.

## 3. Results

The study ultimately included 50 patients. Thirty-five patients had single focus, and 15 patients had multiple focus. A total of 67 TAAs, 67 ANHPs, and 38 TNAs were selected. There were 52 tumors located in the right lobe of the liver, 13 in the left lobe, and 10 in the junction between the left and right lobes. The size of the lesions ranged from 1.8–14.6 cm, and the average diameter was 6.3 ± 3.6 cm (Table 2). Of the 50 participants, the procedure was elective TACE in 2 patients and super-elective TACE in 48 patients.

### 3.1. Intraclass Correlation Coefficients (ICCs) between the Two Readers

The ICCs were assessed for the TAA, TNA, and ANHP measurements. The results showed an excellent agreement (between 0.770 and 0.960). The details are shown in Table 3.

### 3.2. Differences in IVIM Parameters between the Three Tissues

**Figure 2:**
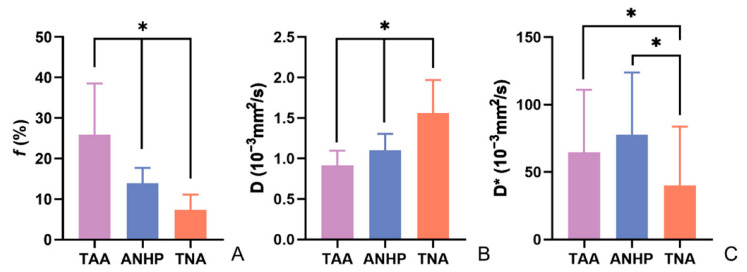
Mean values of various parameters of TAA, ANHP, and TNA. (**A**–**C**) The mean values of parameters *f*, D, and D* respectively. TAA: tumor active area; ANHP: adjacent normal hepatic parenchyma; TNA: tumor necrotic area. *f*: perfusion fraction; D: pure diffusion coefficient; D*: pseudo-diffusion coefficient. * represents that there is an obvious difference (*p* < 0.05).

**Figure 3:**
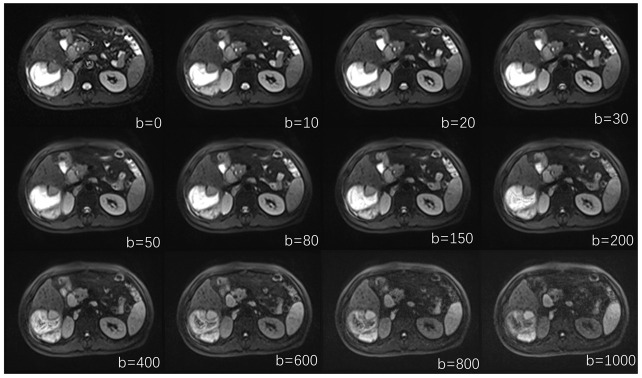
IVIM images at different b values. IVIM-DWI images with 12 b-values, representing b values of 0, 10, 20, 30, 50, 80, 150, 200, 400, 600, 800, and 1000 s/mm^2^.

**Figure 4:**
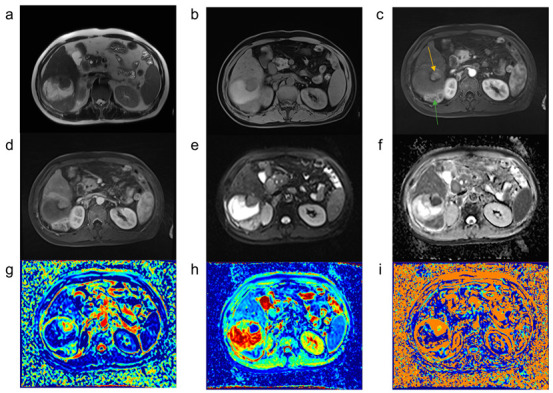
Magnetic resonance images of hepatocellular carcinoma treated using TACE. Most of the tumors after TACE treatment were necrotic, with the tumor active area (orange and green arrows) visible on the edge, which was significantly enhanced at the arterial phase, and the DWI image also showed a high signal intensity. (**a**) T2-weighted image; (**b**) T1-weighted image; (**c**,**d**) MR enhanced images; (**e**) diffusion-weighted image with a b value of 0 s/mm^2^; (**f**) ADC image; (**g**–**i**) parametric maps (*f*, D, and D*, respectively) calculated from the IVIM.

The parameter values of the different tissues and their differences are shown in Table 4 and Figure 2. The f value of the TAAs (25.96 ± 12.58%) was significantly higher than that of the ANHPs (13.88 ± 3.78%, *p* < 0.05) and TNAs (7.41 ± 3.72%, *p* < 0.05); D value of TAAs (0.91 ± 0.18 × 10^−3^ mm^2^/s) was significantly less than that of ANHPs (1.10 ± 0.20 × 10^−3^ mm^2^/s, *p* < 0.05) and TNAs (1.56 ± 0.41 × 10^−3^ mm^2^/s, *p* < 0.05). Figure 3 and Figure 4 show typical images of the IVIM maps. 

### 3.3. Parametric Diagnostic Capabilities among Different Tissues

**Figure 5:**
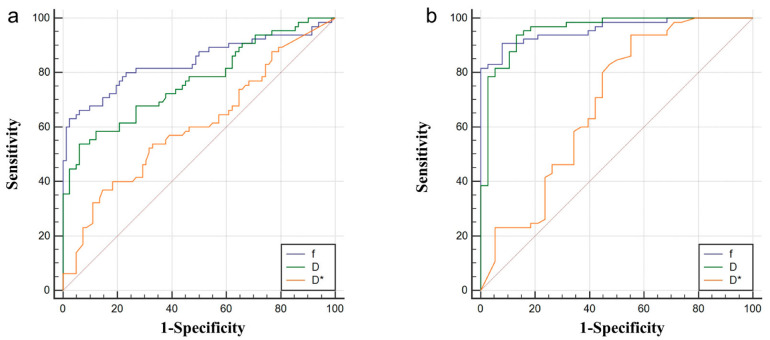
The *f*, D, and D* identify ROC curves of TAA from ANHP (**a**) and TAA from TNA (**b**). TAA: tumor active area; ANHP: adjacent normal hepatic parenchyma; TNA: tumor necrotic area.

Table 5 shows the diagnostic capability of each parameter of IVIM among different tissues. From the receiver operating characteristic (ROC) curve, it can be seen that the values of *f* and D have good diagnostic abilities for TAA and ANHP (areas under the ROC curve (AUCs): 0.835 and 0.753, respectively). The discrimination capacity of the D* value was low for distinguishing TAA and ANHP (AUC 0.591). All of the parameters indicated a good specificity. The parameters f and D exhibited high competence for distinguishing TAA and TNA (AUC: 0.959 and 0.955, respectively) and a showed high sensitivity and specificity. D* had a poor discrimination between TAA and TNA (AUC 0.692) (Figure 5).

## 4. Discussion

Le Bihan first proposed the concept of IVIM in brain magnetic resonance application research [20]. We considered the role of IVIM without a contrast agent for evaluating HCC tumors after TACE in this study. We found that the IVIM quantitative parameters *f* and D played an important role in identifying different tissues after TACE for HCC. These parameters could be used to discriminate TAA, TNA, and ANHP and to supplement quantitative information for the MRI evaluation of HCC after TACE.

We found that the *f* value could effectively distinguish different tumor tissues. If the *f* value was more than 19.81%, it was more likely to be TAA. The *f* value represents the percentage capillary volume in the entire tissue volume of the voxel, which is related to the microvessel capillary density. Whether the capillaries are normal or immature, the *f* value reflects the speed of angiogenesis and the extent of blood perfusion to a certain extent. TACE treatment significantly inhibits or eliminates the tumor activity and causes total or partial tumor necrosis. The microvessel density of the tumor necrotic tissue was significantly reduced, which was reflected through a distinct decline in *f* value. The microvessel density of the adjacent normal liver parenchyma was between that of the active tumor tissue and necrotic tumor tissues, so its *f* value was greater than that of the necrotic tissue but lower than that of the tumor tissue.

Parameter D indicates the dispersion state of water molecules in the tissue (in mm^2^/s). In this study, if the D value was less than 0.91 × 10^−3^ mm^2^/s, it was more likely to be TAA. The active area of the tumors was relatively small and the activity of the water molecules was relatively limited, while the D value was relatively low. However, in the area of tumor necrosis, after interventional treatment, the tumor cells were destroyed and divided, the intercellular space increased, and the movement of water molecules was not restricted, so the D value increased. Our study demonstrates that the parameter of D was the highest in TNA and lowest in TAA.

The IVIM-DWI parameters were not only valuable for differentiating TAA, TNA, and ANHP, but also had significance for the evaluation of the TACE treatment effect in liver cancer and for prognostic determination. Park YS et al. used IVIM scanning to predict the effect of lipiodol deposition in TACE. They divided patients into a good lipiodol intake (LGU) group and a poor lipiodol intake (LPU) group, and found that the value of D in the LGU group was superior to the value in the LPU group. These results indicate that the IVIM parameters could help predict the lipiodol intake [21]. Studies have shown that the values of *f* and D are significant parameters for evaluating the effect of sorafenib on advanced liver cancer [22,23]. Lifang Wu et al. believed that the D ratio was an independent predictor of progression-free survival outcomes for liver cancer after TACE [24].

Fifty participants in this study had the TACE procedure after MR examination without surgical resection, and thus had no pathological findings. LI-RADS TRA is a widely used method for assessing the locoregional treatment response in clinic. Kim et al. [25] evaluated the viable tumors with a specificity of 98% for both reviewers based on Gd-EOB-MRI images. Jae Seok Bae et al. [26] confirmed that the specificity of hepatobiliary agent-enhanced MRI using the LI-RADS treatment response algorithm (TRA) to assess viable was 93.9%. Yoon et al. [27] showed that the specificity of evaluation as LR-TR viable after transarterial radioembolization treatment was 93.3–100%, and of the lesions evaluated as nonviable with LR-TR, as 80.0% (12 of 15) were completely necrotic histopathologically. In Mohammad Chaudhry’s study [28], 15 lesions were evaluated as LR-TR viable by LIRADS TRA, 11 of which showed a viable tumor upon histopathology; 32 lesions were classified as LR-TR nonviable, 26 of which were completely necrotic upon histopathology. Those results showed that when the LI-RADS treatment response was evaluated as viable, it was relatively consistent with the pathological results. When the LI-RADS treatment response was evaluated as nonviable, most MR evaluation results corresponded to the pathology, but a few were inconsistent with the pathology. We finally combined the LI-RADS and DSA results to make the results more accurate.

After TACE, the tumor-feeding vessels are embolized, and the tumor becomes ischemic and hypoxic, eventually leading to cellular necrosis, which is manifested in the IVIM sequence by a gradual decrease in *f*-values and a gradual increase in D-values. The tumor-necrotic effect of TACE may be manifested clinically by fever and pain and an increase in transaminases, and studies have shown that the increase in transaminases after TACE is mainly secondary to tumor necrosis, and that transient post-treatment transaminase elevation may predict an objective response to superselective cTACE in clinical practice [29].

The lower b-value images could afford a higher signal-to-noise ratio, mainly reflecting information related to blood perfusion, but the sensitivity of identifying molecular diffusion to diffusion secondary to microcirculation was reduced. The higher b value tends to be in the straight line mode, which could almost completely eliminate diffusion after the microcirculation, reflecting the true diffusion parameter value; thus, the signal-to-noise ratio may be reduced, leading to a poor image quality [30]. In terms of b-value selection, there is currently no unified international standard, and most researchers set different b-value ranges according to their experimental purposes. Studies have shown that different b-value selections have a certain impact on the results. A reasonable multiple b-value selection could improve the accuracy of the results [31] and reduce the error, but this would increase the scanning time. In the 0–50 s/mm^2^ range, more b-values could enhance the precision of the *f* and D* values and reflect the real perfusion situation. We comprehensively considered the scan time and the accuracy of the data, selected 12 b-values and set most of the b-values within the scope of 0–50 s/mm^2^ to achieve a total scan time for this group of patients of approximately 20 min, which most patients can tolerate.

Our research was limited to a single-center study, resulting in a relatively small number of patients. A large-scale multicenter study could provide additional data to support our conclusions. Second, the patients in our study did not have pathological results after TACE, so our gold standard was mainly based on TACE retreatment results and LI-RADS standards, which can guarantee the accuracy of the lesion analysis. In the follow-up study, we will focus on collecting patients who have undergone surgical resection after TACE, and analyze and discuss the pathological results.

## 5. Conclusions

In conclusion, IVIM, especially parameter *f,* plays an active role in the quantitative evaluation of the treatment response of hepatocellular carcinoma after TACE and may be an alternative non-invasive method to monitor the tumor viability in patients with HCC after TACE.

## Figures and Tables

**Table 1:** MR imaging sequence parameters.

Sequence	Orientation	TR (ms)	TE (ms)	FOV (mm)	Slice Thickness (mm)
T2 -weighted HASTE	Axial	1000	95	380 × 380	6
T1 -weighted FLASH	Axial	6.91	2.39	380 × 380	3
Diffusion-weighted imaging	Axial	4100	58	380 × 380	6
IVIM-DWI	Axial	4900	54	380 × 380	3
Radial VIBE	Axial	3.57	1.69	380 × 380	5

HASTE: half-Fourier-acquired single-shot turbo spin echo; FLASH: fast low-angle shot; TE: echo time; TR: repetition time; FOV: field of view.

**Table 2:** Patient and lesion characteristics on IVIM.

Characteristics	N
Sex	
Women	9
Men	41
Age (y)	54 ± 10
Etiology of HCC	
Hepatitis B virus	40
Cryptogenic	8
Hepatitis C virus	2
Alcoholism	1
Cirrhotic	
Yes	41
No	9
Child-Pugh class *	
Child-Pugh A	41
Child-Pugh B	8
AFP ^#^	
Normal	11
Abnormal	35
Number of HCC lesions per patient	
Multiple	15
Single	35
Number of ROIs	
ROIs of the tumor active area	67
ROIs of the tumor necrotic area	38
ROIs of the adjacent normal hepatic parenchyma	67

Data are described as the number of patients, lesions, and mean ± standard deviation. * One of the patients lacked the information regrding the Child-Pugh class. ^#^ Four of the patients lacked the information regarding the AFP. HCC: hepatocellular carcinoma; ROIs: regions of interest; AFP: alpha-fetoprotein.

**Table 3:** ICC of the values between the two readers.

Parameters	TAA (95%CI)	ANHP (95%CI)	TNA (95%CI)
*f*	0.850 (0.766–0.905)	0.826 (0.732–0.889)	0.869 (0.762–0.930)
D	0.901 (0.844–0.938)	0.870 (0.796–0.918)	0.960 (0.925–0.979)
D*	0.892 (0.830–0.933)	0.865 (0.789–0.915)	0.770 (0.599–0.873)

TAA: tumor active area; TNA: tumor necrotic area; ANHP: adjacent normal hepatic parenchyma; *f*: perfusion fraction; D: pure diffusion coefficient; D*: pseudo-diffusion coefficient.

**Table 4:** The parameter values of different tissues and their differences.

Parameters	TAA (*n* = 67)	TNA (*n* = 38)	ANHP (*n* = 67)	*p*
*f* (%)	25.96 ± 12.58 *&	7.41 ± 3.72 #	13.88 ± 3.78	<0.05
D (10^−3^ mm^2^/s)	0.91 ± 0.18 *&	1.56 ± 0.41 #	1.10 ± 0.20	<0.05
D* (10^−3^ mm^2^/s)	64.66 ± 46.42 &	40.04 ± 43.66 #	77.52 ± 46.32	<0.05

* represents that there is an obvious difference between TAA and ANHP (*p* < 0.05); # represents that there is an obvious difference between TNA and ANHP (*p* < 0.05); & represents that there is an obvious difference between TAA and TNA (*p* < 0.05). Data are described as the mean ± standard deviation. TAA: tumor active area; TNA: tumor necrotic area; ANHP: adjacent normal hepatic parenchyma; *f*: perfusion fraction; D: pure diffusion coefficient; D*: pseudo-diffusion coefficient.

**Table 5:** Diagnostic capability of IVIM parameters for distinguishing different tissues.

.	Parameter	AUC (95%CI)	Youden Index	Sensitivity	Specificity	Threshold Value
TAA and ANHP	*f*	0.835 (0.761–0.894)	0.5970	62.69%	97.01%	19.81%
	D	0.753 (0.671–0.823)	0.4478	52.24%	92.54%	0.91 (×10^−3^ mm^2^/s)
	D*	0.591 (0.503–0.675)	0.1940	31.34%	88.06%	27.32 (×10^−3^ mm^2^/s)
TAA and TNA	*f*	0.959 (0.902–0.988)	0.8315	91.04%	92.11%	12.32%
	D	0.955 (0.896–0.986)	0.7973	95.52%	84.21%	1.19 (×10^−3^ mm^2^/s)
	D*	0.692 (0.594–0.778)	0.3877	94.03%	44.74%	15.90 (×10^−3^ mm^2^/s)

TAA: tumor active area; ANHP: adjacent normal hepatic parenchyma; TNA: tumor necrotic area. *f*: perfusion fraction; D: pure diffusion coefficient; D*: pseudo-diffusion coefficient.

## Data Availability

The detailed data presented in the study are included in the article, and further inquiries can be directed to the corresponding author.
